# Percutaneous endoscopic interlaminar discectomy for posterior ring apophyseal fracture accompanied with lumbar disc herniation in a 12-year pediatric diver: a case report

**DOI:** 10.1007/s00381-022-05605-5

**Published:** 2022-07-07

**Authors:** Hui Wu, Sikuan Zheng, Dingwen He, Xigao Cheng

**Affiliations:** Department of Orthopaedic Surgery, The Second Affiliated Hospital of Nanchang University, Nanchang, 330006 Jiangxi China

**Keywords:** Pediatric, Posterior ring apophysis fracture

## Abstract

Posterior ring apophysis fracture (PRAF), accompanied with lumbar disc herniation (LDH), is a rare occurrence. Owing to its rarity, there is no consensus on the treatment strategy for this condition. Differences mainly encompass the type of decompression method, the need for additional spinal fusion, the need for apophysis fragments or/and disc materials removal, and long-term efficacy, particularly, compared to LDH alone. Hence, the aim of this study was to describe a rare instance of PRAF with LDH in a 12-year-old professional diver, who was successfully treated with percutaneous endoscopic interlaminar discectomy (PEID), and to initiate a discussion involving several meaningful and related factors.

## Introduction

Posterior ring apophysis fractures (PRAF), accompanied with lumbar disc herniation (LDH), is relatively uncommon [1]. Severe PRAF will increase the risk of spinal deformity in growing children [2]. Due to the persistence of bone compression, the outcome of conservative treatment is often poor [3]. When conservative therapy is considered ineffective, surgical intervention is advocated [3]. The goal of a PRAF with LDH-correcting operation is to relieve symptoms, with minimal surgical trauma, while enabling the patient to rapidly return to normal physical activity [3, 4]. However, the unique physiological characteristics of pediatric patients mandate the PRAF treatment to possess certain distinctive characteristics, and be more minimally invasive. Therefore, here, we report a rare case of PRAF with LDH in a 12-year-old professional diver, who was successfully treated with PEID.

## Case presentation

**Fig. 1:**
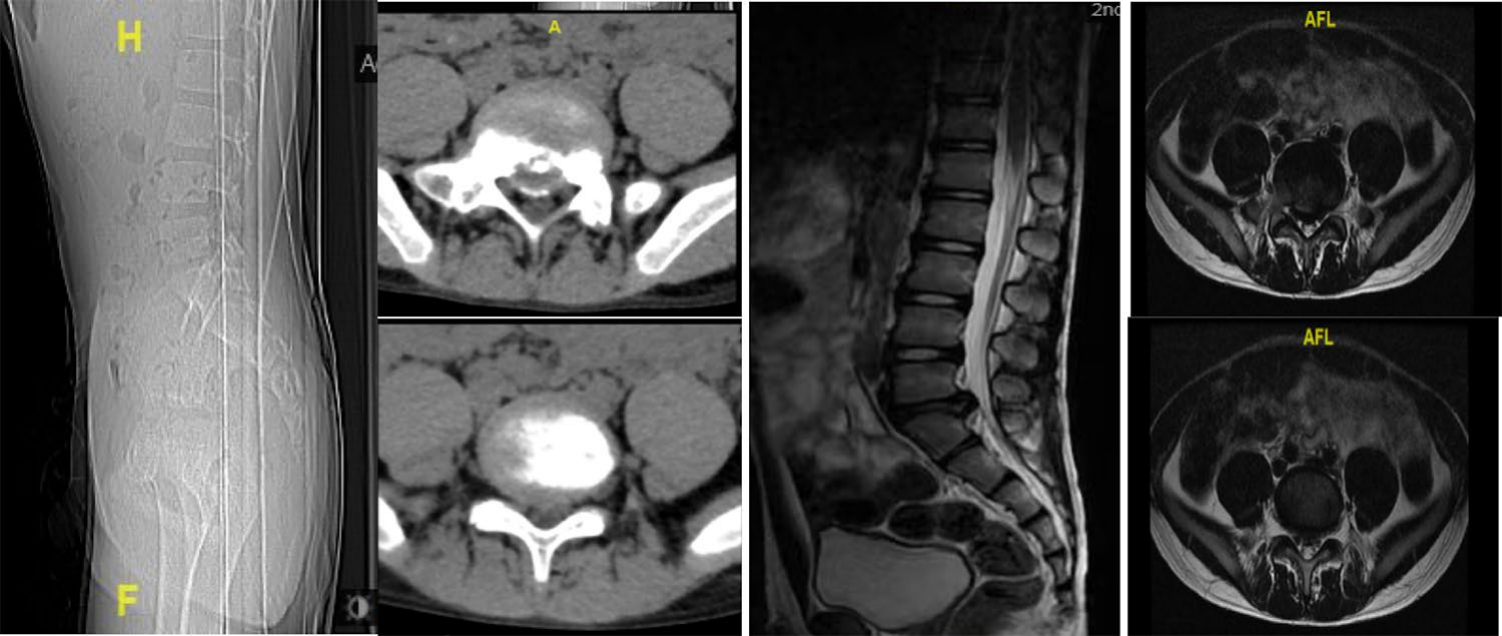
The preoperative imaging demonstrated obvious L5/S1 level ring apophysis fractures with LDH

A 12-year-old male professional diver suffered from lower back pain and right lower limb radiculopathy for over 9 months. Apart from his father, who experienced LDH, there were no other diseases reported in the family. Physical examination: The right-straight-leg-raise test was positive and muscle strength of the right lower limb decreased slightly. The preoperative imaging demonstrated obvious L5/S1 level ring apophysis fractures with LDH (Fig. 1).

## Surgical intervention

**Fig. 2:**
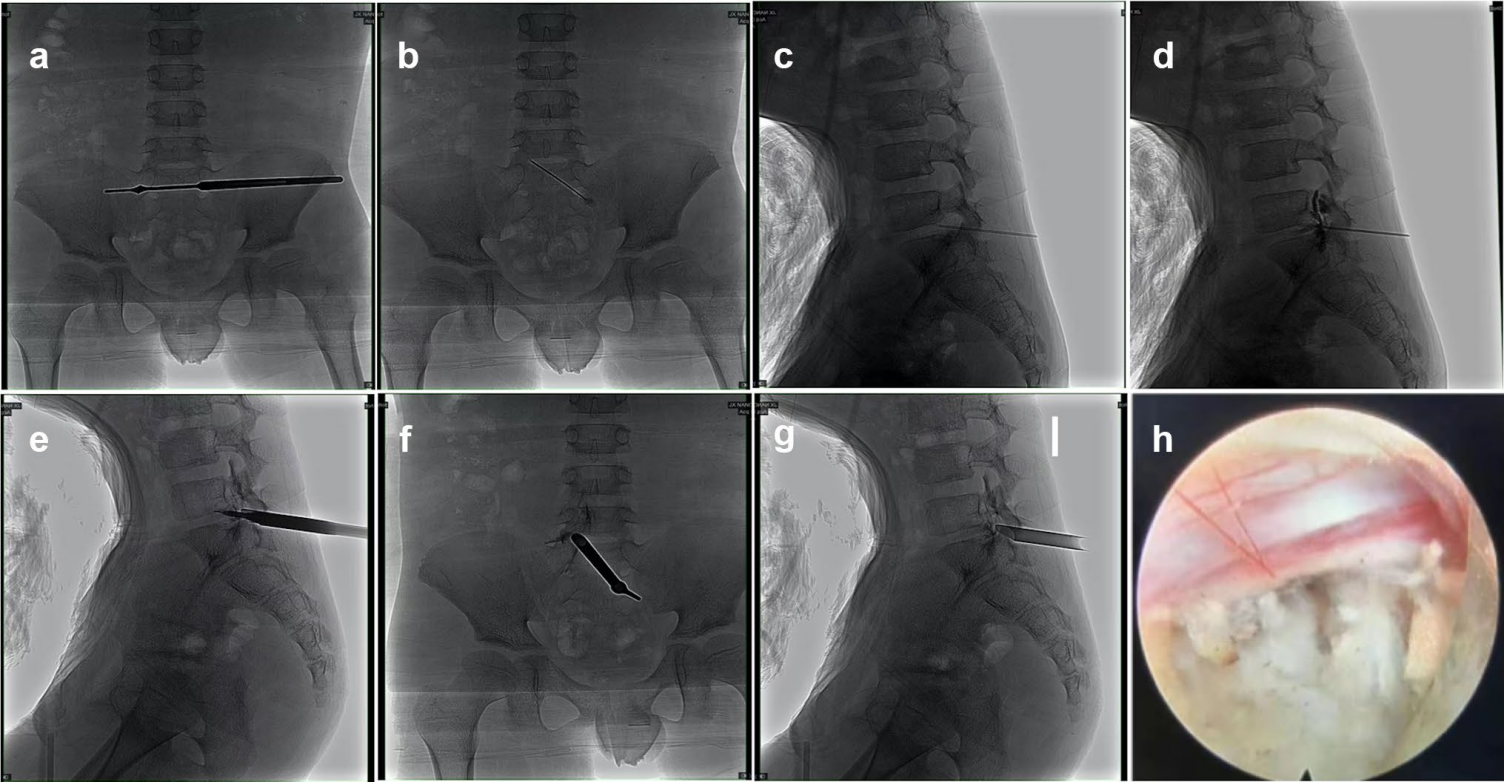
Confirm the operative segment (**a**). The standard position of needle tip (**b**, **c**). Inject the contrast medium (**d**). Break through the surface of the ligamentum flavum (**e**). Final position of working channel (**f**, **g**). Traversing nerve root was visualized (**h**)

**Fig. 3:**
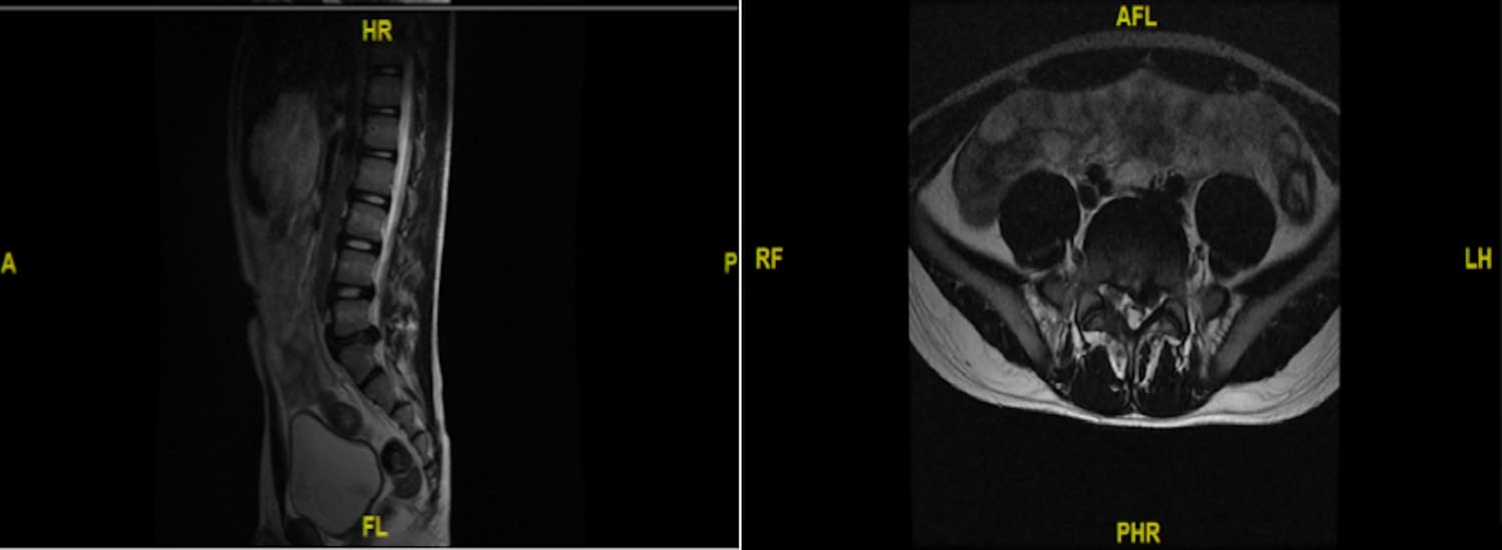
Asymptomatic residual disc tissue was observed on MRI 3 months after operation

The patient was placed in a prone position on a radiolucent operating table and was provided with local anesthesia. The C-arm X-ray fluoroscopy was used to confirm the operative segment (Fig. 2a). The 18 G needle trajectory was introduced with its tip pointed at the lateral edge of the interlaminar window (Fig. 2b, c). Once the puncture needle reached the appropriate location, the contrast medium was injected (Fig. 2d). The needle was then removed, with a 1-mm-diameter guide wire inserted, and a dilator was introduced with its tip puncturing through the surface of the ligamentum flavum (Fig. 2e). A beveled working sheath was then inserted through the dilator with its beveled opening toward the spinal canal (Fig. 2f, g). Subsequently, an endoscope was introduced through the working cannula. Different types of forceps were employed for decompression, and then the traversing nerve root was visualized (Fig. 2h). The shoulder and axillary sides of the nerve roots were explored, prior to the removal of the working channel and endoscope. The patient’s straight-leg-raise test value increased from 50 to 90° after PEID. Although asymptomatic residual disc tissue was noted on magnetic resonance imaging (MRI) at the 3-month follow-up post operation (Fig. 3), the patient was able to return to his original high level of activity, without any residual lower back pain at the 6-month follow-up post operation. No complications were observed during the follow-ups.

## Discussion and conclusions

PRAF is a rare etiology of back pain in adolescents and young adults, and it is, unfortunately, ignored by many surgeons [1, 3]. Trauma is considered a significant causative factor in about 50% of children and adolescents [1, 5]. The main pathogenesis of the presented case included ring apophysis, and incompletely fused vertebral endplate following high-intensity dive training. PRAF mainly occurs at the L4-5 and L5-S1 disc levels [1, 3, 5]. PRAF diagnosis primarily depends on computed tomography (CT), and it can be classified into four sub-groups [3, 6]. Early surgical intervention is advocated for patients with persistent symptoms. Laminectomy or semi-laminotomy, along with discectomy via the posterior approach, is a strongly recommended surgical procedure [3]. Different approaches are chosen to reach this principle, such as classical microdiscectoms [3], tubular microdiscectomy (TMD) [7], and percutaneous endoscopic discectomy (PED) [6]. The surgical trauma and dual scars caused by classical microdiscectomy are the greatest challenges for patients at the present time [6]. TMD can achieve more extensive bone resection, which is especially applicable to the central fragment fractures (i.e., types I, II, and IV), which can eventually lead to either spinal canal or intervertebral foramen stenosis [3, 6]. Our patient presented with a unilateral lesion and was classified as a type III. Hence, a unilateral semi-laminotomy or laminectomy could have achieved satisfactory results [6]. PEID was previously reported as a treatment option for type III PRAF in young adults, and it possesses the characteristics of markedly reduced epidural scar, iatrogenic injury, and surgical trauma [6]. Therefore, PEID offers more advantages in treating type III PRAF than TMD. It is not mandatory to remove the separated fragments because there is no difference in clinical results [8]. Most authors recommended that herniated discs must be removed [9, 10]. However, few authors suggested that discectomy must not be performed in pediatric patients, particularly, if they do not display degenerative disc disease [3]. Our recommendation was that discectomy must be considered, based on age and MRI findings. Therefore, limited nucleus pulposus removal was performed in our patient. Zheng et al. reported that over 90% of PRAF patients achieve good or excellent results at the 2-year follow-up after PEID [6]. However, few scholars are attentive to the surgical effect of PRAF, accompanied with LDH, relative to LDH alone. Following a comprehensive review of available literature, we discovered that patients with PRAF, accompanied with LDH, exhibit equal long-term clinical prognoses and postoperative complications, compared to LDH patients alone [8, 11].

In summary, PEID is an optimal approach for treatment of PRAF with LDH (type III) in a pediatric diver.

## Data Availability

The original contributions generated for the study are included in the article/Supplementary Material, and further inquiries can be directed to the corresponding author.

## Abbreviations

PRAF: Posterior ring apophysis fracture
LDH: Lumbar disc herniation
PEID: Percutaneous endoscopic interlaminar discectomy
MRI: Magnetic resonance imaging
CT: Computed tomography
TMD: Tubular microdiscectomy
PED: Percutaneous endoscopic discectomy
